# The Specifically Androgen-Regulated Gene (SARG) Promotes Papillary Thyroid Carcinoma (PTC) Lymphatic Metastasis Through Vascular Endothelial Growth Factor C (VEGF-C) and VEGF Receptor 3 (VEGFR-3) Axis

**DOI:** 10.3389/fonc.2022.817660

**Published:** 2022-06-13

**Authors:** Shuai-Jun Xu, Bin Jin, Wei-Jun Zhao, Xue-Xian Chen, Ying-Ying Tong, Xiao-Fei Ding, Ying-Yuan Chen, Dong-Hao Wang, Zhi-Ming Wang, Bing-Qing Dai, Sai Chen, Yong Liang, Guang Chen, Su-Jiao Pan, Ling-Long Xu

**Affiliations:** ^1^ Department of Hematology, Taizhou Central Hospital (Taizhou University Hospital), Taizhou University, Taizhou, China; ^2^ Graduate School of Medicine, Hebei North University, Zhangjiakou, China; ^3^ Department of Clinical Medicine , School of Medicine, Taizhou University, Taizhou, China; ^4^ Department of Pharmacology, Shenyang Pharmaceutical University, Shenyang, China; ^5^ Department of Pharmacology, School of Medicine, Taizhou University, Taizhou, China; ^6^ Department of Pathology, Women’s Hospital of Jiaojiang Districts, Taizhou, China

**Keywords:** papillary thyroid carcinoma (PTC), lymphatic metastasis, specifically androgen-regulated gene (SARG), vascular endothelial growth factor, VEGF-C

## Abstract

The papillary thyroid carcinoma (PTC) metastasizes through lymphatic spread, but the follicular thyroid cancer (FTC) metastasis occurs by following hematogenous spread. To date, the molecular mechanism underlying different metastatic routes between PTC and FTC is still unclear. Here, we showed that specifically androgen-regulated gene (SARG) was significantly up-regulated in PTC, while obviously down-regulated in FTC through analyzing the Gene Expression Omnibus (GEO) database. Immunohistochemistry assay verified that the PTC lymph node metastasis was associated with higher levels of SARG protein in clinical PTC patient samples. SARG-knockdown decreased TPC-1 and CGTH-W3 cells viability and migration significantly. On the contrary, SARG-overexpressed PTC cells possessed more aggressive migratory ability and viability. *In vivo*, SARG overexpression dramatically promoted popliteal lymph node metastasis of xenografts from TPC-1 cells mouse footpad transplanting. Mechanistically, SARG overexpression and knockdown significantly increased and decreased the expression of vascular endothelial growth factor C (VEGF-C) and VEGF receptor 3 (VEGFR-3), respectively, thereby facilitating or inhibiting the tube formation in HUVECs. The tube formation experiment showed that SARG overexpression and knockdown promoted or inhibited the number of tube formations in HUVEC cells, respectively. Taken together, we showed for the first time the differential expression profile of SARG between PTC and FTC, and SARG promotes PTC lymphatic metastasis *via* VEGF-C/VEGFR-3 signal. It indicates that SARG may represent a target for clinical intervention in lymphatic metastasis of PTC.

## Introduction

Thyroid cancer is the most common endocrine malignancy, and its global incidence increases year by year due to heavy use of diagnostic imaging and surveillance (1, 2). Differentiated thyroid cancer (DTC) including papillary thyroid carcinoma (PTC) and follicular thyroid cancer (FTC) accounts for more than 90% of all thyroid cancers (3). They tend to be easier to treat than other types but display different routes of metastasis.

PTC is the most common subtype of thyroid cancer with the best overall prognosis, accounting for approximately 70% of cases, although 15% PTCs show higher aggressiveness and poorer outcomes (4). Of PTC patients 20%-90% occur in regional lymph node (LN) metastasis and 10%-15% of patients appear as distant metastasis, which negatively impacts the overall survival (5). Unlike PTC, which tends to metastasize to lymph nodes, FTC tends to metastasize to remote organs especially lungs and bones by the hematogenous route (6). However, the mechanism of different metastatic pathways between PTC and FTC is still unclear.

The specifically androgen-regulated gene (SARG) protein is located in the cytoplasm and has a length of 601 amino acids with two isoforms as the result of alternative splicing. The expression of SARG is up-regulated by androgen, but not by glucocorticoids (7). There are few reports about its function now because the amino acid composition of SARG does not indicate motifs that could predict its function. Parsana et al. (8) discovered SARG was a novel epithelial to mesenchymal transition (EMT) gene whose decreased expression is associated with poor prognosis in lung and prostate cancer patients. However, whether and how the mechanism of SARG regulates the progression of thyroid cancer is currently unclear.

In the present study, we explored the expression profile of SARG between PTC and FTC, then further investigated its roles in PTC metastasis occurring by following lymphatic spread.

## Materials and Methods

### GEO Data

GSE 33630 and GSE82208 Series were downloaded from the GEO database (http://www.ncbi.nlm.nih.gov/geo/) including 45 normal thyroid tissues, 49 papillary thyroid carcinoma, and 27 follicular thyroid cancer tissues. The differentially expressed genes (DEGs) were performed using the edgeR package in the R language (https://bioconductor.org/packages/edgeR/), setting P-value < 0.01 and log2 |FC| > 1 as the threshold.

### Cell Culture and Reagents

CGTH-W3 and TPC-1 cells were obtained from Taizhou Central Hospital. 293T human kidney epithelial cell line was purchased from the Chinese Academy of Sciences Type Culture Collection (CASTCC). CGTH-W3 cells were maintained in RPMI 1640 medium supplemented with 10% fetal bovine serum (FBS) while TPC-1 and HEK-293T cells were grown in DMEM containing 10% FBS. Cells were cultured at 37°C in a humidified incubator with 95% air and 5% CO_2_. The polybrene was purchased from MCE (Shanghai, China). The pLenti-EF1a-EGFP-P2A-Puro-CMV-SARG-Flag plasmid was designed and was synthesized from OBiO Biotechnology Corp, Ltd. (Shanghai, China). The pMDG2.G and psPAX2 plasmids for lentivirus assembling were obtained from Addgene.

### Immunohistochemistry

Thyroid samples of patients were collected by the Taizhou Central Hospital. Immunohistochemistry was performed as follows. Sample sections were dewaxed with xylene, hydrated with ethanol, then run through an antigen repair protocol. Endogenous peroxidases were neutralized by 0.3% H_2_O_2_ in dH_2_O for 10 min. After washing, slides were blocked with 10% ready-to-use goat serum for 0.5 h at room temperature. Tissue sections were incubated with the primary antibodies(SARG, Proteintech; VEGF-C, Proteintech; VEGFR3, Proteintech)overnight. After washing with PBS, samples were incubated with HRP anti*-*rabbit IgG (1:200) at room temperature for 30 min and diaminobenzidine (DAB) was used as a sensitive chromogen and then counterstained with hematoxylin. Slides were analyzed by taking photos with a microscope (BX53, Olympus, Japan).

### Lentivirus-Mediated Transfection

For lentivirus transfection, pLenti-CMV-SARG plasmids (2.5 μg) with a mixture of packaging plasmids (0.75 μg pMD2.G, 1.90 μg psPAX2) were co-transfected into HEK293T cells by using Lipofectamine 2000 (Invitrogen). Infection efficiency was evaluated by observing the percentage of the green fluorescent protein (GFP) expression by inverted fluorescence microscope. The viral supernatant was collected 48-72 h post-transfection. Viral supernatant supplemented with polybrene 8 μg/mL (Sigma-Aldrich) was added to cells. Forty-eight hours after the addition of viruses, SARG-GFP-infected cells were selected with puromycin (MCE). The expression of SARG was confirmed by real-time qPCR and Western blotting.

### Transfection of siRNA

The synthetic siRNAs targeting VEGF-C and negative control were obtained from Gene Pharma. Sequences are as follows: 5’-GCCGAUGCAUGUCUAAACUTT-3’ (VEGF-C); 5’-UUCUCCGAACGUGUCACGUTT-3’ (negative control). Transfection is at 60-70% cell density using Lipofectamine 2000 (Invitrogen) with a final siRNA concentration of 100 nM.

### Real-Time qPCR

Total RNAs were harvested by using the Trizol reagent according to the manufacturer’s instructions, then reverse transcription was performed by the reverse transcription kit (Takara Biotechnology Co., Ltd.) to obtain the cDNA. The synthetized cDNA was amplified by real-time qPCR using a SARG-specific primer, CAGTCTCAACCAGGTACACAC (forward), TAG TCGGCTGTTTGGGTCCT (reverse); VEGF-C primer, GGCTGGCAACATAACAGAGAA (forward), CCCCACATCTATACACACCTCC (reverse); VEGFR3 primer, AGGGAGACGCCCTTTCATG (forward), GAGGGCTCTTTGGTCAAGCA (reverse); GAPDH primer, GCACCGTCAAGGCTGAGAAC (forward), GCCTTCTCCATGGTGGTGAA (reverse). Real-time qPCR cycles were the following: 95°C for 30 s (step 1), 40 cycles of 95°C for 5 s, 60°C for 30 s (step 2), and then 95°C for 15 s, 60°C for 60 s, and 95°C for 15 s (step 3). The target gene expression was calculated by the 2−ΔΔCt method with the following formula: ΔΔCt=ΔCt sample−ΔCt control gene wherein ΔCt =Ct target gene − Ct internal reference.

### Western Blotting

Cells were harvest with RIPA lysis solution (Beyotime, China). The total protein concentration was quantified by the BCA method (Thermo Fisher Scientific, Inc.). Western blotting (immunoblotting) was conducted in accordance with a standard experimental procedure. Total protein was separated by 10% SDS-polyacrylamide gel and transferred onto PVDF membranes. The membranes were blocked with 5% nonfat dry milk in TBST (40 mM Tris–HCl, 0.5 M NaCl, 0.1 Tween-20, pH 7.4) at room temperature for 2 h. Primary antibodies (SARG, Proteintech; VEGF-C, Proteintech; VEGFR-3, ABACM; ERK1/2, Proteintech; Phosphorylated-p44/42 ERK1/2, Cell Signaling Technology; GAPDH, Santa Cruz Biotechnology) were diluted in the antibody diluent at 4°C overnight, and secondary antibodies (anti-rabbit 1:10000 and anti-mouse 1:10000) were diluted in TBST for 1 h at room temperature. The detection and quantification of protein bands were captured with the Image Quant LAS 4000 Mini Imager (GE Healthcare Bio-Sciences).

### Cell Viability

Cell viabilities were measured using the MTT assay. Cells were digested for single-cell suspensions and seeded in 96-well plate with a density of 1000 cells per well. Four compound holes were set for each group. After culturing for 4 h, 24 h, 48 h, 72 h, and 96 h, 10 μL MTT (5 mg/ml) reagent was added into each well and incubated at 37°C for 4 h. Then, the supernatant fractions were removed and 150 μL dimethyl sulfoxide (DMSO) was added to each well to solubilize the crystals. Absorbance at OD 490 nm was recorded.

### Cell Migration Assay

Cell migration was investigated with a pore size of 8 μm Transwell system (Costar). Cells were resuspended into single cells in serum-free medium and cell density was adjusted to 5 × 105 cells/mL. There was 100-µl single cell suspension added to the upper chamber, while in the lower chamber, 500-µL culture medium with 10% FBS was added. After incubation for 12 h at 37°C, cells in the lower chamber were fixed by 70% methanol and stained with 0.1% crystal violet. A total of 5 fields of view were randomly chosen and the numbers of migrated cells were counted under a microscope.

### Popliteal Lymph Node Metastasis Assay

All animal experiments were conducted following appropriate guidelines. The present study was ethically approved by the Medical Ethics Committee of Taizhou University College of Medicine. Female BALB/c nude mice (4 to 5 weeks old) were purchased from Beijing Vital River Laboratory Animal Technology (Hangzhou, China). The mice were randomly divided into 2 groups of 5 mice each in a cage. A total of 10 female mice weighing 15-20 g were housed in sterile cages at 20°C under a 12:12 h light–dark cycle. All experiments were performed according to the Institutional Ethical Guidelines. Lentivirus-transduced parallel-controlled or SARG-overexpressed TPC-1 cells (2 × 10^6^ cells) stably expressing GFP were inoculated into the mouse footpads. After 5 weeks, the fluorescence signals of the primary tumors and the popliteal lymph nodes were detected by an *in vivo* imaging system (Aniview 100, Biolight Biotechnology, Guangzhou). Another 3 weeks later, the popliteal lymph nodes were harvested and fixed in formalin. Tissues were dehydrated and embedded in melted paraffin wax, the resulting block was mounted on a microtome and cut into thin slices. The slices were affixed to microscope slides at which point the wax was removed with a solvent and the tissue slices attached to the slides were rehydrated and were ready for H&E staining.

### Tube Formation Assay

The 48-well plates were coated with 100 μL Matrigel (BD Biosciences, US) per well and maintained at 37°C for 30 min, then Lentivirus-transduced parallel-controlled or SARG-overexpressed HUVEC cells 4×10^4^ were added to wells which were re-suspended in 100 μL complete DMEM medium and incubated at 37°C for 4 or 6 h. Fluorescence microscopy (IX73 Olympus, Japan) was applied to collect the photos with cellSens Standard 1.9 software used to measure the numbers of tubule branches.

### Statistical Analysis

All statistical analyses were carried out in GraphPad Prism version 8.0 (GraphPad Software, Inc.) Statistical significance was tested by Student’s t-test between =2 groups. Chi-squared test was used to determine whether there is a statistically significant difference in popliteal lymph node metastasis status between the control and SARG overexpressed cells. Results were shown as Mean ± Standard Deviation (Mean ± SD). Differences were considered statistically significant at * P < 0.05 and ** P < 0.01.

## Results

### SARG Expression Levels Were Upregulated in PTC and Associated With Lymph Node Metastasis Status

**Figure 1:**
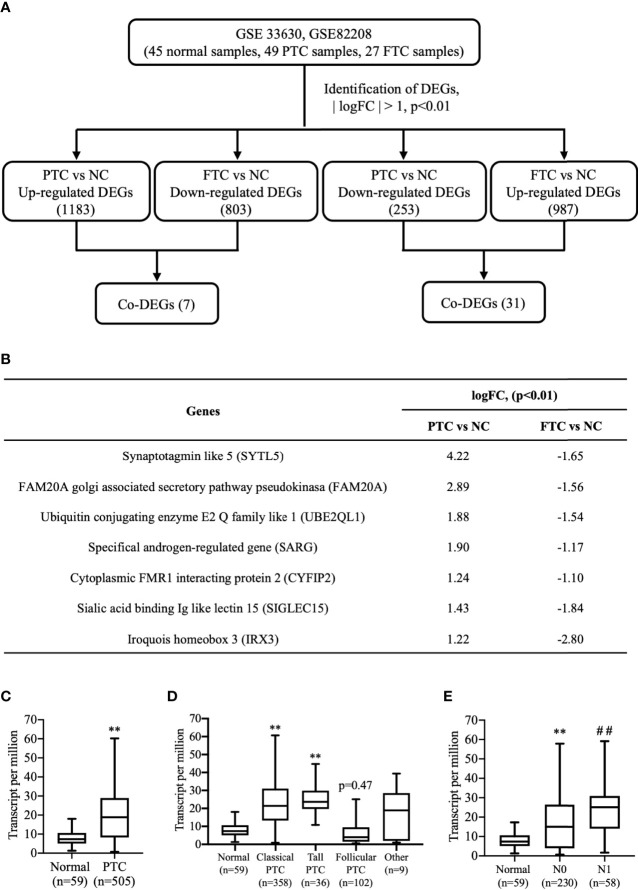
Finding SARG overexpression associated with lymph node metastasis of PTC by analysis of GEO and TCGA database. **(A)** Brief scheme for screening the DEGs between PTC and FTC by GEO database analysis. **(B)** The list of 7 genes is up-regulated in PTC and down-regulated in FTC. **(C)** The expression of SARG between PTC and normal thyroid tissue from TCGA database. **, p<0.01 compared with Normal. **(D)** Expression of SARG in PTC based on tumor histology. **, p<0.01 compared with Normal. **(E)** Expression of SARG in PTC based on nodal metastasis status, **, p<0.01 compared with Normal; ^##^p<0.01 compared with N0, N0: no regional lymph node metastasis, N1: thyroid cancer with lymph node metastases in the axillary region. SARG, specifically androgen-regulated gene; PTC, papillary thyroid carcinoma; GEO, Gene Expression Omnibus; TCGA, The Cancer Genome Atlas; FTC, follicular thyroid carcinoma; DEGs, differentially expressed genes.

To explore the expression profile of SARG between PTC and FTC, we downloaded GSE 33630 and GSE82208 Series with the same platform using Affymetrix Human Genome U133 Plus 2.0 Array. There were 45 normal thyroid tissues, 49 papillary thyroid carcinoma, and 27 follicular thyroid cancer tissues included in the GEO database to perform the edgeR package. mRNAs showing log2|FC|>1 and p<0.01 were considered differentially expressed genes by following the analysis schedule as shown in **Figure 1A**. Through a comparison of the gene mRNA levels in cancer and normal tissues, 1183 DEGs and 987 DEGs were screened out with higher mRNA levels in PTC and FTC, respectively, than in normal tissues, while 803 genes and 253 genes expressed lower levels in PTC and FTC, respectively, compared to normal tissues (**Figure 1A**). We further screened out 7 DEGs showing increased profiles in PTC while decreased expression in FTC including SARG. The gene list is shown in **Figure 1B**.

TCGA database analysis confirmed most types of PTCs showed higher levels of SARG than normal thyroid tissues (**Figure 1C**), and more interestingly, even follicular papillary thyroid carcinoma expressed lower SARG mRNA levels (**Figure 1D**). Furthermore, expression of SARG in PTC based on nodal metastasis status in TCGA database analysis showed that PTC with higher SARG levels was associated with more aggressive nodal metastasis (**Figure 1E**).

**Figure 2:**
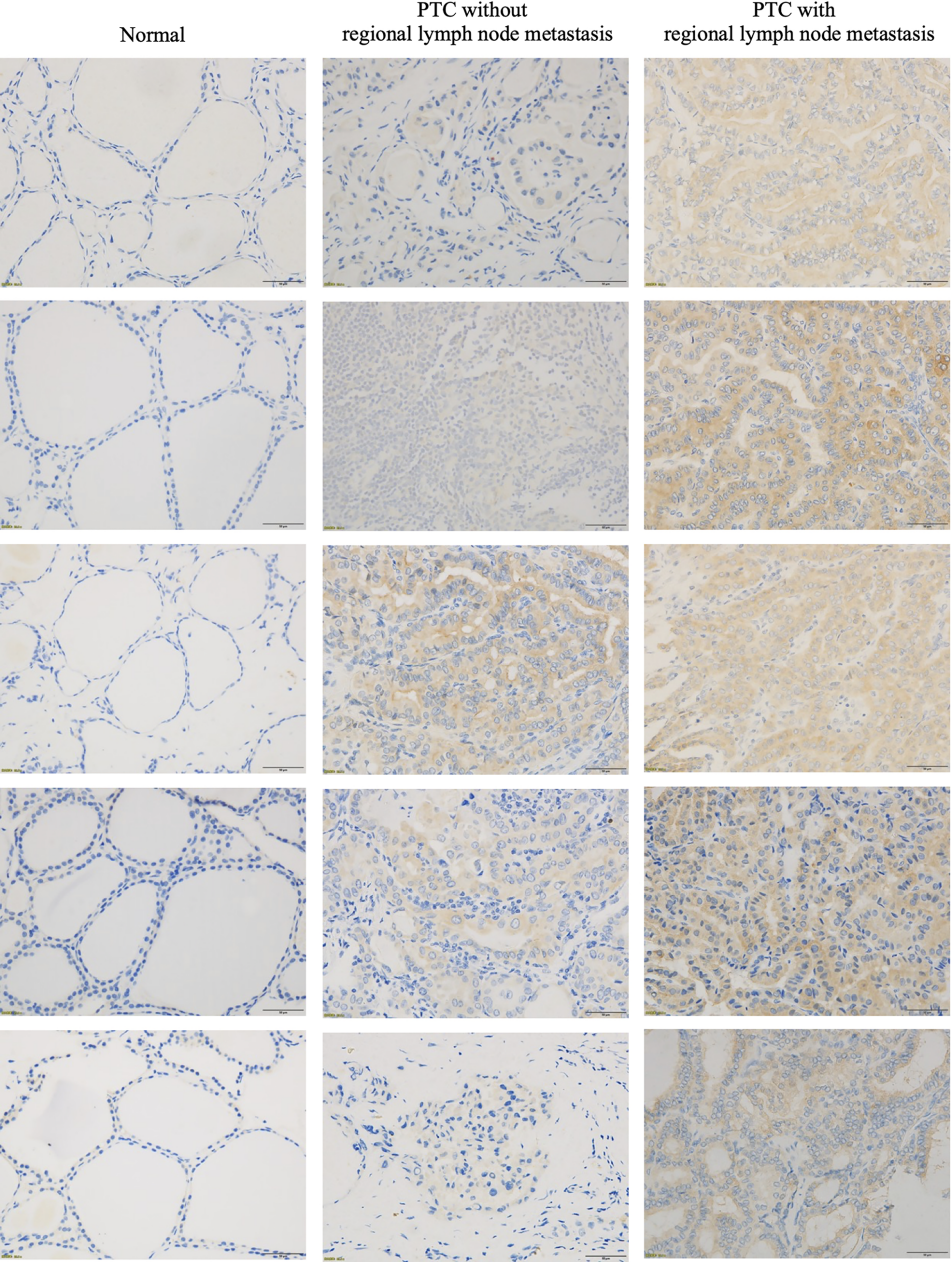
Representative immunohistochemistry assay images of SARG staining in thyroid normal tissues, PTC tissue without regional lymph node metastasis, and PTC tissue with lateral cervical lymph node metastasis. SARG, specifically androgen-regulated gene; PTC, papillary thyroid carcinoma.

To determine whether SARG was involved in the regulation of lymph node metastasis of PTC, we evaluated the differential expression profile of SARG between tissue specimens with positive lymph node metastasis and tissue specimens with negative lymph node metastasis by immunohistochemistry. The results showed that SARG exhibited higher expression in lymph node-positive tissue specimens than that in lymph node-negative tissue specimens (**Figure 2**). Altogether, overexpression of SARG may exert crucial effects on the growth and progression of PTC.

### SARG Overexpression Promoted Cell Viability and Migration in TPC-1 and CGTH-W3 Cells

**Figure 3:**
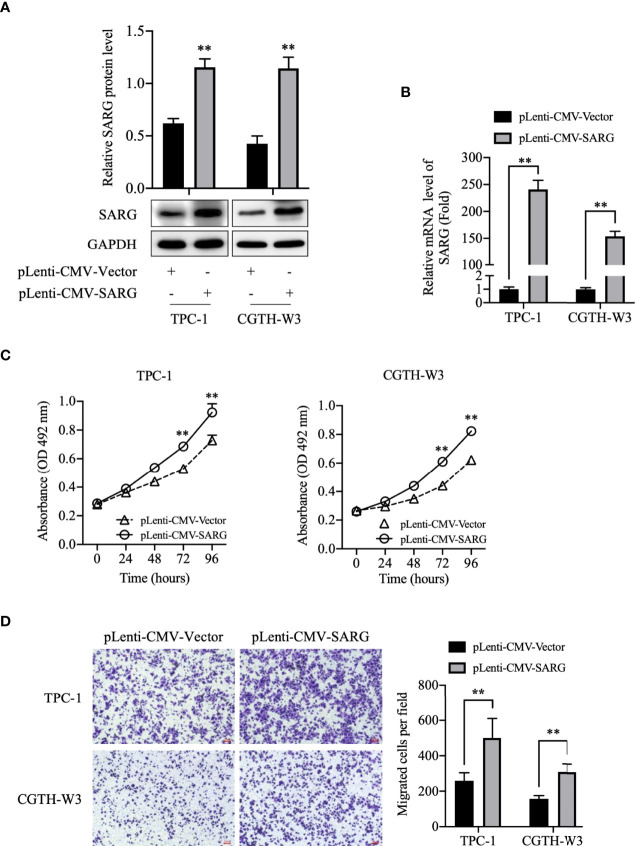
SARG overexpression promoted PTC cells proliferation and migration. **(A)** SARG protein expression in TPC-1 and CGTH-W3 cells was significantly increased after being transfected with pLenti-CMV-SARG plasmid. GAPDH was used as a loading control, whereas pLenti-CMV-vector was used as the negative control. **(B)** SARG overexpression efficiency was evaluated by reverse transcription-quantitative PCR, with all expression levels normalized to those of GAPDH. Data are presented as fold changes relative to the SARG levels in control cells. **(C)** SARG overexpression could increase TPC-1 and CGTH-W3 cells proliferation. **(D)** SARG overexpression promoted TPC-1 and CGTH-W3 cells migration in transwell assay. SARG, specifically androgen-regulated gene. **, p<0.01 pLenti-CMV-Vector.

To better investigate the role of SARG in PTC progression, we constructed SARG-overexpressed cell lines using the lentivirus system in TPC-1 and CGTH-W3 cells that were PTC cell lines. As shown in **Figures 3A, B**, SARG protein and mRNA levels were increased obviously in TPC-1 and CGTH-W3 cells transfected with pLenti-CMV SARG. Consequently, cells transfected with pLenti-CMV SARG grew faster than control cells in MTT assay (**Figure 3C**). Furthermore, the results of transwell migration assay showed that SARG overexpression could raise cell migration in TPC-1 and CGTH-W3 cells (**Figure 3D**).

### SARG Knockdown Inhibited Cell Viability and Migration in TPC-1 and CGTH-W3 Cells

**Figure 4:**
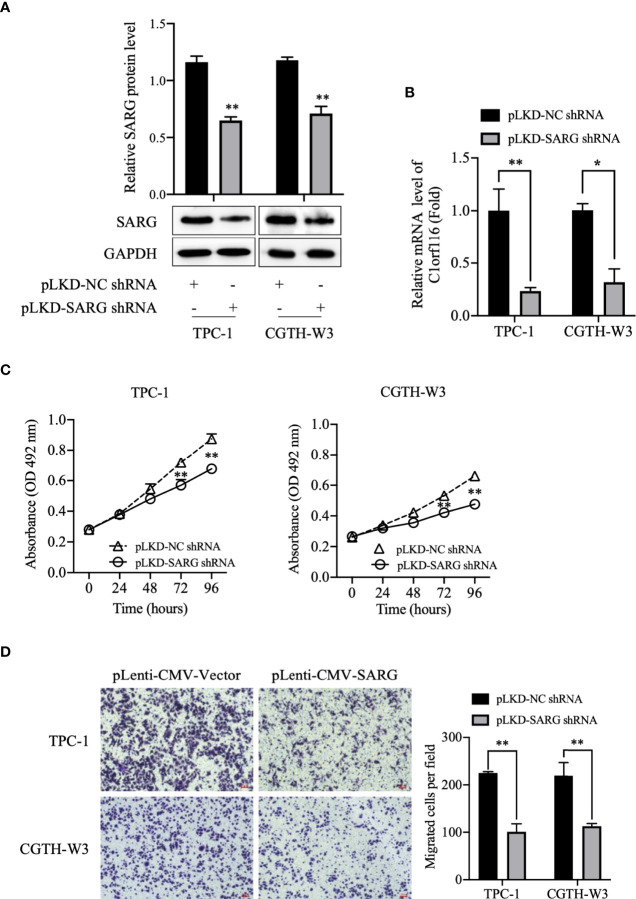
SARG knockdown impaired PTC cells proliferation and migration. **(A)** SARG protein expression in TPC-1 and CGTH-W3 cells was significantly decreased after being transfected with SARG-shRNA. GAPDH was used as a loading control, whereas NC-shRNA was used as the negative control. **(B)** SARG knockdown efficiency was evaluated by reverse transcription-quantitative PCR, with all expression levels normalized to those of GAPDH. Data are presented as fold changes relative to the SARG levels in control cells. **(C)** SARG knockdown could reduce TPC-1 and CGTH-W3 cells proliferation. **(D)** SARG knockdown inhibited TPC-1 and CGTH-W3 cells migration in transwell assay. SARG, specifically androgen-regulated gene. *, p<0.05, **, p<0.01 compared with NC shRNA.

To further determine the role of SARG in PTC progression, TPC-1 and CGTH-W3 thyroid cancer cells lines were transfected with shRNA targeting SARG using the lentivirus system to knockdown SARG. As shown in **Figures 4A, B**, the Western blot and real-time qPCR results showed that SARG-shRNA transfection significantly down-regulated the protein and mRNA level of SARG. MTT cell viability assay data indicated that down-regulation of SARG significantly inhibited cell survival in TPC-1 and CGTH-W3 cells (**Figure 4C**). Transwell cell migration assay results showed that down-regulation of SARG reduced the cell migration in TPC-1 and CGTH-W3 cells (**Figure 4D**).

### SARG Overexpression Promoted Popliteal Lymph Node Metastasis of PTC Cells Footpad Xenografts in Mice

**Figure 5:**
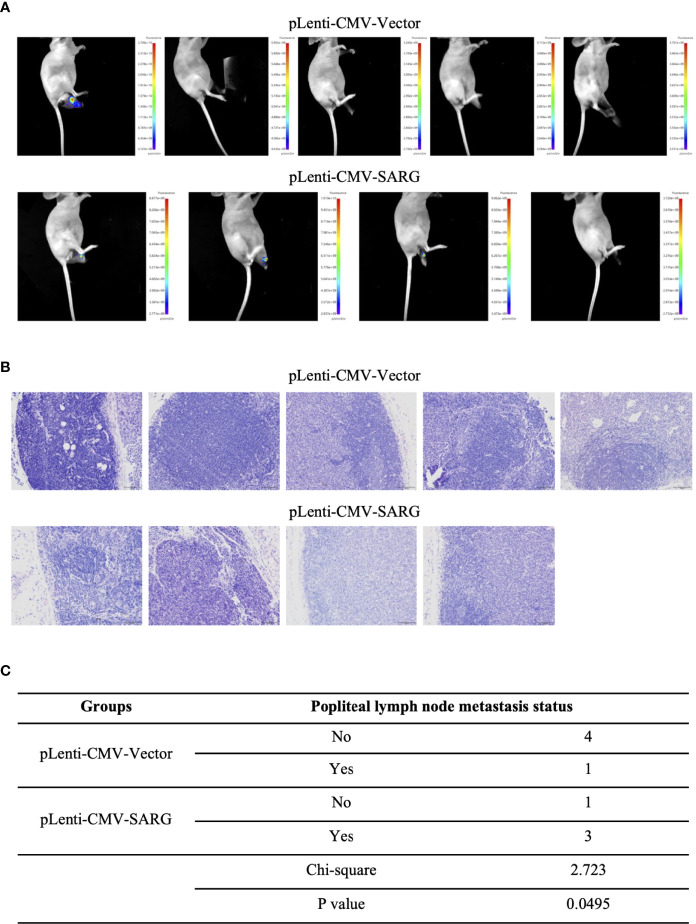
SARG overexpression promoted popliteal lymph node metastasis of PTC cells footpad xenografts in mice. **(A)** TPC-1 cells overexpressed SARG were injected subcutaneously into the footpads of nude mice, 5 weeks later, lymphatic spread was detected by bioluminescence imaging. **(B)** Hematoxylin and eosin staining of popliteal lymph node at the termination. Scale bars, 100 µm. **(C)** χ^2^ test of the popliteal lymph node metastasis rate.

To further validate the role of SARG in PTC metastasis, *in vivo* experiments were performed. TPC-1 cells overexpressing SARG were injected subcutaneously into the footpads of nude mice. As expected, in comparison to mouse xenografts bearing TPC-1 cells transfected with control vectors, xenografts bearing TPC-1 cells overexpressing SARG showed grossly lymphatic spread being detected by an *in vivo* imaging system as early as 5 weeks later after cell injection (**Figure 5A**). Meanwhile, metastasis in the lymph nodes of mice bearing PTC cells was apparent in H&E staining of lymph node tissue sections (**Figure 5B**). A total of 75% popliteal lymph nodes in mice bearing SARG overexpressing xenografts contain tumor cells, whereas the rate of metastasis to popliteal lymph node was only 20% in mice bearing pLenti-CMV vector plasmid xenografts. There was a significant difference between the vector control and SARG overexpressed group in the popliteal lymph node metastasis rate based on the χ^2^ test (**Figure 5C**).

### SARG Was Involved in VEGF-C and VEGFR-3 Regulation

**Figure 6:**
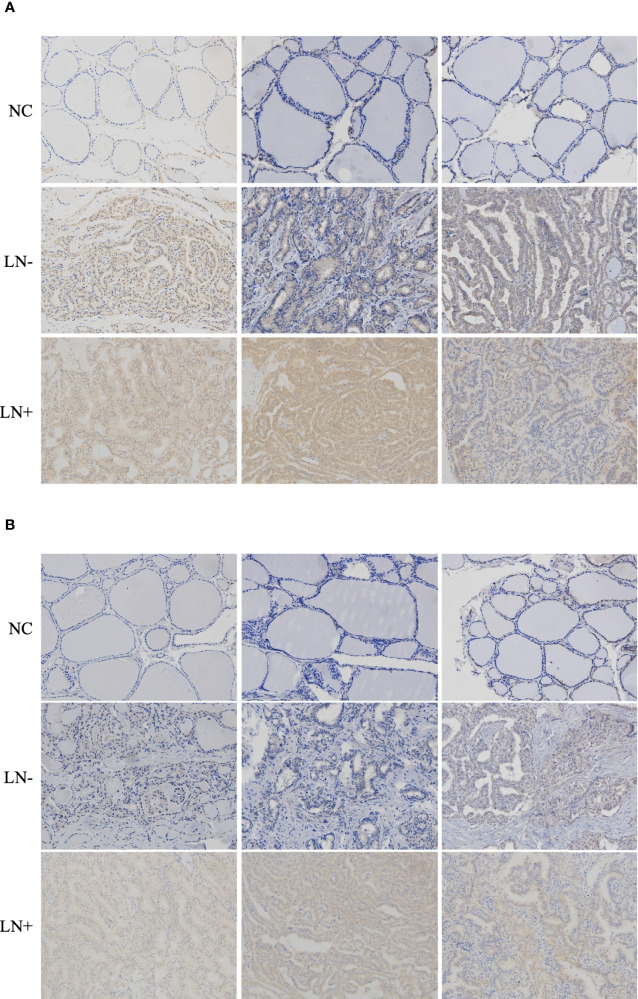
Representative immunohistochemistry assay images of VEGF-C **(A)** and VEGFR3 **(B)** staining in thyroid normal tissues, PTC tissue without regional lymph node metastasis, and PTC tissue with lateral cervical lymph node metastasis. VEGF-C, vascular endothelial growth factor C; VEGFR-3, vascular endothelial growth factor receptor 3; PTC, papillary thyroid carcinoma.

**Figure 7:**
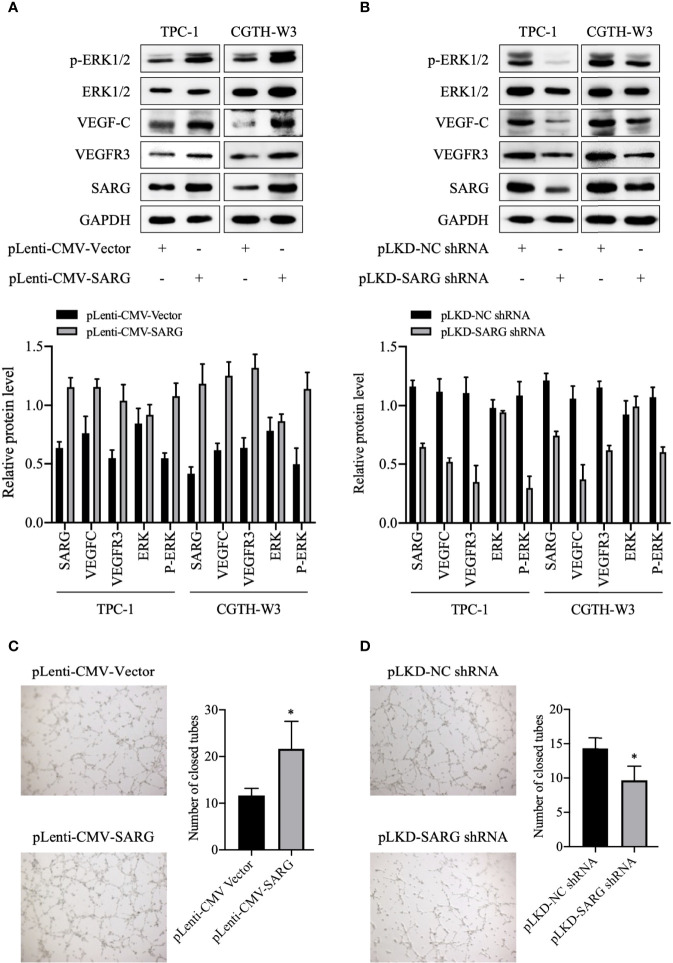
SARG was involved in VEGFC/VEGFR3 regulation and HUVEC tube formation. **(A, B)** VEGF-C and VEGFR3 levels were analyzed by immunoblotting upon SARG overexpression or knockdown, with GAPDH used as the loading control. **(C, D)** The tube formation ability of HUVECs was detected by tube formation assay. * p<0.05 compared with NC-shRNA. SARG, specifically androgen-regulated gene; VEGF-C, vascular endothelial growth factor C; VEGFR-3, vascular endothelial growth factor receptor 3.

**Figure 8:**
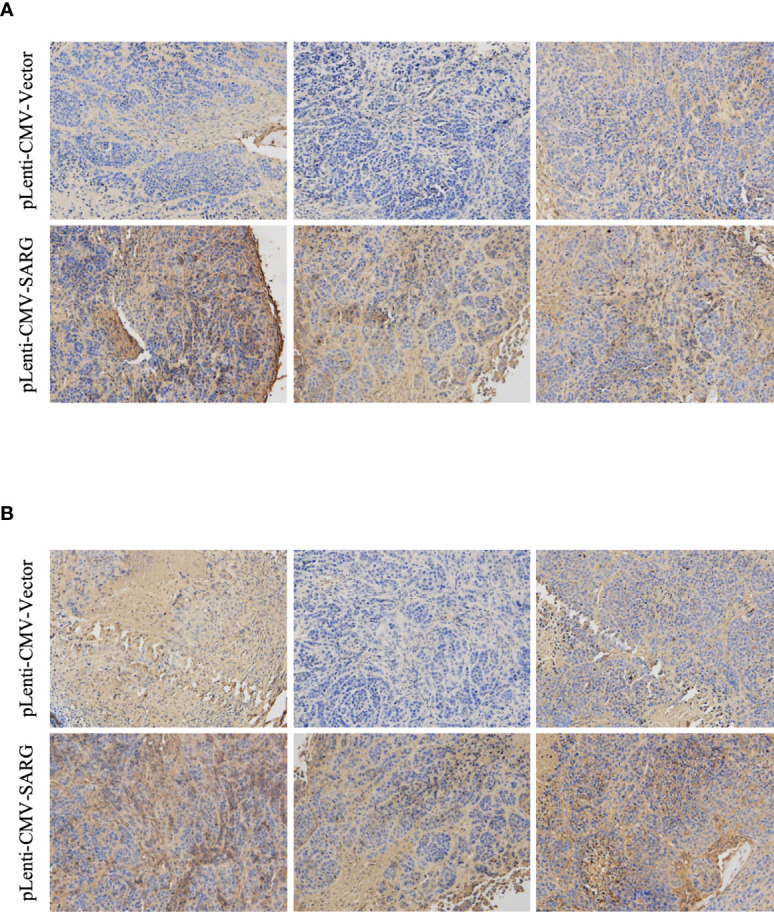
Representative immunohistochemistry assay images of VEGF-C **(A)** and VEGFR3 **(B)** staining in TPC-1 xenografts tissue transfected with vector control plasmids or SARG overexpressing plasmids. VEGF-C, vascular endothelial growth factor C; VEGFR-3, vascular endothelial growth factor receptor 3; SARG, specifically androgen-regulated gene.

VEGF-C/VEGFR3 axis has been recognized as a critical regulator of lymph node metastasis (9). We also confirmed that VEGF-C and VEGFR3 exhibited higher expression in PTC tissue compared to normal thyroid tissue, and it was highly expressed in lymph node-positive tissue specimens than in lymph node-negative tissue specimens (**Figure 6**). To explore the molecular mechanisms underlying the role of SARG in PTC metastasis, VEGF-C/VEGFR-3 protein levels were measured upon SARG overexpression or knockdown. Western blotting analysis revealed that SARG overexpression increased VEGF-C and VEGFR-3 protein levels in TPC-1 and CGTH-W3 cells (**Figure 7A**). Whereas SARG ablation drastically suppressed the expression of VEGF-C and VEGFR-3 (**Figure 7B**). Furthermore, immunohistochemical experiments were used to detect VEGF-C and VEGFR3 in all the primary xenografts of nude mice. As shown in **Figure 8**, VEGF-C and VEGFR3 expression were upregulated in the SARG overexpressed group than that in the vector control group.

A previous study indicated that VEGF-C stimulated lymphatic endothelial cells migration by activating both ERK pathways (10). VEGF-C binds to VEGFR3 and then activates the ERK pathway, which is critical for endothelial and cancer cell survival and progression. Therefore, we observed how the phosphorylated ERK1/2 protein changed after up-regulation or knock-down of SARG. As shown in **Figure 7A**, the protein level of phosphorylated ERK1/2 was increased in SARG-overexpressing TPC-1 and CGTH-W3 cells, while knockdown of SARG showed the contrary results (**Figure 7B**). Tube formation assays were then performed to assess whether SARG could regulate lymphangiogenesis in HUVECs. As shown in **Figure 7C**, SARG over-expression notably promoted lymphangiogenesis in HUVECs. We could find about 12 closed tubes in the vector control group cells under microscope, while there were about 21 closed tubes in SARG overexpressed cells. On the contrary, SARG knockdown dampened lymphangiogenesis (**Figure 7D**).

**Figure 9:**
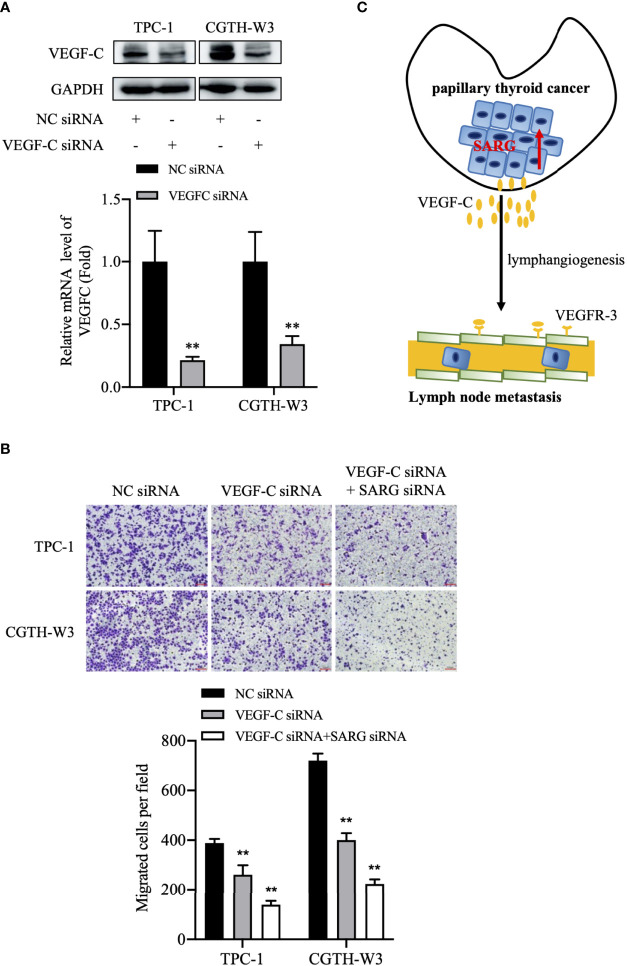
The putative model of lymphatic metastasis mediated by SARG in PTC. **(A)** VEGF-C protein expression in TPC-1 and CGTH-W3 cells was significantly decreased after being transfected with VEGF-C siRNA. GAPDH was used as a loading control, whereas NC siRNA was used as the negative control. **(B)** Knocking down VEGF-C promoted then anti-migration activity of SARG-siRNA in PTC cells. ** p<0.01 compared with NC-siRNA. C, SARG was upregulated in PTC and could upregulate VEGF-C expression in PTC cells, then promoted lymphangiogenesis and lymph node metastasis. PTC, papillary thyroid carcinoma; SARG, specifically androgen-regulated gene; VEGF-C, vascular endothelial growth factor C.

We test the change of SARG function after knockdown of VEGFC. As shown in **Figures 9A, B**, SARG-siRNA enhances VEGF-C siRNA mediated migration down-regulation.

## Discussion

In the present study, we found 7 DEGs showing increased profiles in PTC while decreased expression in FTC by analyzing GEO database. Previous preliminary experiments found that down-regulation of SARG would reduce the expression of VEGFR3 in PTC cells, suggesting that SARG may play a role in PTC lymphatic metastasis. To date, there is no published research article about its roles in thyroid cancer. We further showed that SARG has a different expression profile between PTC and FTC by analyzing GEO and TCGA database. We observed here higher levels of SARG in PTC samples with regional lymph node metastasis than those without regional lymph node metastasis. SARG knockdown inhibited PTC cell viability and migration *in vitro*, while its overexpression promoted PTC cell viability, migration *in vitro*, and popliteal lymph nodes metastasis in mice footpad xenografts model. Our primary data provide a new point to study the molecular mechanisms whereby SARG modulates lymphatic metastasis of PTC.

Due to the wide application of diagnostic imaging and fine-needle aspiration biopsy in thyroid nodules, the incidence of thyroid cancer, a common endocrine tumor, has gradually increased in recent years (11). There are 567,000 cases of thyroid cancer worldwide, ranking ninth in incidence (12). Papillary thyroid carcinoma is the most common histological subtype. Although PTC is generally considered to be an indolent tumor, some patients have central lymph node metastasis and cervical lymph node metastasis. Patients with lymph nodes tend to have a high recurrence rate and low survival rate (13). The molecular mechanism of PTC lymph node metastasis is still unclear. In this study, we analyzed the GEO database and found that the expression of SARG was in up-regulated PTC, while down-regulated in FTC. In IHC assay, there were higher levels of SARG in PTC samples with regional lymph node metastasis than those without regional lymph node metastasis, which indicates that SARG might be involved in the regulation of PTC metastasis *via* lymphatic spread.

SARG was first found highly expressed in prostate cancer, its down-regulation induced epithelial to mesenchymal transition in epithelial prostate cancer cell line PC3 cells, suggesting that it may be a new EMT regulatory factor (8). However, there are no reports about its role in other tumors, including thyroid cancer. Here, we showed that SARG knockdown reduced TPC-1 and CGTH-W3 cells viability and migration *in vitro*. On the contrary, SARG overexpression promoted the PTC cells viability and migratory ability in TPC-1 and CGTH-W3 cells by MTT and transwell assay *in vitro*, and the growth of footpad PTC xenograft and popliteal lymph node metastasis in nude mice. Overall, these findings indicate that SARG accelerates tumorigenesis and lymph node metastasis of PTC.

Lymph angiogenesis, an early metastatic event, is considered a necessary condition for lymph node metastasis and is a powerful prognostic indicator for patients with thyroid cancer (14, 15). In addition, lymphangiogenic growth factors and receptors are essential for regulating the mechanism of lymph angiogenesis. Cancer cells secrete lymphangiogenic growth factors to promote the spread of tumor cells to lymph nodes and induce the formation of new lymphatic vessels (16). VEGF-C/D and VEGFR3 signaling has been found to be the most important pathway of lymph angiogenesis (17). VEGF-C first induces the activation of VEGFR3, which leads to the phosphorylation of serine kinases ERK, and ultimately promotes the proliferation, migration, and survival of lymphatic endothelial cells (18). VEGF-C is a lymphatic vessel-specific growth factor, which is increased in expression in various human tumors, including patients with lymph node metastasis of thyroid cancer (19, 20). Here, we show that SARG overexpression or knockdown increases or decreases VEGF-C and VEGFR3 levels, respectively, in PTC cells. Our study shows for the first time that SARG may act by regulating the VEGF-C/VEGFR3 axis in thyroid cancer cells.

In summary, we show SARG is differently expressed in between PTC and FTC tissues. SARG promotes PTC lymphatic metastasis by regulating VEGF-C/VEGFR3 axis (**Figure 9C**). Additional molecular research is needed to understand how SARG regulates VEGF-C and VEGFR3.

## Data Availability Statement

The original contributions presented in the study are included in the article/supplementary material. Further inquiries can be directed to the corresponding authors.

## Ethics Statement

The present study was ethically approved by the Medical Ethics Committee of Taizhou University College of Medicine.

## Author Contributions

S-JX, GC, YL, S-JP, L-LX and SC performed data analysis, data interpretation, and wrote the manuscript; X-FD, B-QD, Z-MW, BJ, X-XC, D-HW, Y-YC, Y-YT, and S-JX conducted the experiment. W-JZ assisted with GEO database analysis. All authors contributed to the article and approved the submitted version.

## Funding

This study was, in part, funded by Public Technology Research Projects of the Science Technology Department of Zhejiang Province (No. LY20H310003, LGF19H050004, and LGD20H310001), the National Natural Science Foundation of China (No. 81802657 and 81201530), Scientific Research Foundation of the Education Department of Zhejiang Province (Y201941713), Medical Health Science and Technology Project of Zhejiang Provincial Health Commission (2020385091), Technology Research Projects of the Science Technology Department of Taizhou (No. 20ywb97, 1901ky77, 1901ky54, and 1902ky45), Medical and Health Technology of Zhejiang Province (No. 2020RC147), and Medical Health Science and Technology Project of Jiangxi Provincial Health Commission (202130717).

## Conflict of Interest

The authors declare that the research was conducted in the absence of any commercial or financial relationships that could be construed as a potential conflict of interest.

## Publisher’s Note

All claims expressed in this article are solely those of the authors and do not necessarily represent those of their affiliated organizations, or those of the publisher, the editors and the reviewers. Any product that may be evaluated in this article, or claim that may be made by its manufacturer, is not guaranteed or endorsed by the publisher.
